# Clear cell variant of calcifying epithelial odontogenic tumor: Case report with immunohistochemical findings 

**DOI:** 10.4317/jced.51995

**Published:** 2015-02-01

**Authors:** Eveline Turatti, Juviano Brasil, Bruno-Augusto-Benevenuto de Andrade, Mário-José Romañach, Oslei-Paes de Almeida

**Affiliations:** 1 DDS, PhD. Department of Oral Pathology, School of Dentistry, University of Fortaleza, UNIFOR; 2 DDS, MSc. Department of Oral Pathology, School of Dentistry, University of Fortaleza, UNIFOR; 3 DDS, PhD. Department of Oral Diagnosis and Pathology, School of Dentistry, Federal University of Rio de Janeiro, UFRJ; 4 DDS, PhD. Department of Oral Diagnosis, Piracicaba Dental School, University of Campinas, FOP-UNICAMP

## Abstract

Calcifying epithelial odontogenic tumor (CEOT) is a rare benign odontogenic neoplasm, locally aggressive, characterized by sheets and nests of polyhedral epithelial cells exhibiting eosinophilic cytoplasm or less often clear cytoplasm. Additional features include nuclear pleomorphism without mitotic activity, concentric calcifications, and deposits of amyloid. Herein, we present an additional example of clear cell variant of CEOT occurring in a 25-year-old female. Microscopically, the tumor consisted on proliferation of epithelial cells with eosinophilic, clear vacuolated cytoplasm interspersed with focal areas of amyloid deposition. Tumor cells were immunopositive for AE1/AE3, CK14, CK19, β-catenin, CD138, and p63.

** Key words:**Calcifying epithelial odontogenic tumor, clear cell, histopathology, immunohistochemistry.

## Introduction

Calcifying epithelial odontogenic tumor (CEOT) is an uncommon benign odontogenic tumor with local invasiveness potential that has been extensively reported in the literature, mainly through single or small series of cases (1-15).

The main clinical and radiographic features include a slow-growing asymptomatic swelling in the posterior mandible of adult patients, presenting as a well-delimited unilocular or multilocular mixed radiolucencent-radiopaque lesion with displacement of bone cortical and teeth (1-3). CEOT is microscopically characterized by cords and nests of round to polygonal eosinophilic cells with nuclear pleomorphism and conspicuous intercellular bridges in a fibrous stroma that typically contains variable amounts of the Congo red-positve amyloid-like material and calcified structures (1-7).

In 1967, Abrams and Howell described the first case of CEOT predominantly composed of clear cells (3). Some years later, Krolls and Pindborg considered two of those 23 cases of CEOT as a diagnostic challenge due their high content of clear cells. Since then, the predominance of the clear cell component in CEOTs has been reported mainly through single cases and its prognostic importance is still debatable (1-8). We report an additional case of clear cell variant of CEOT with immunohistochemical findings.

## Case Report

**Figure 1:**
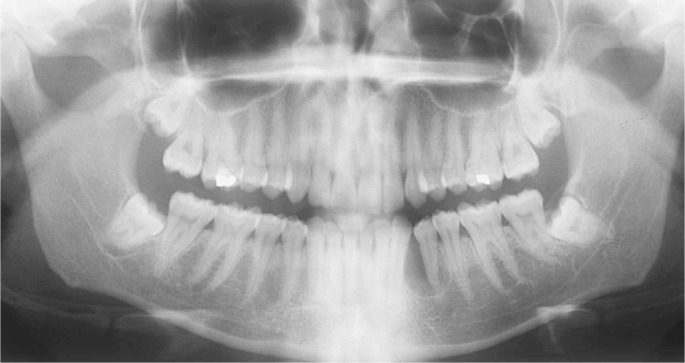
Panoramic radiography of clear cell variant of calcifying epithelial odontogenic tumor. A well-delimited radiolucency measuring 2 x 1,5 cm was identified between the lower second premolar and canine of the left side. The root of canine was displaced anteriorly and there is no dental resorption.

**Figure 2:**
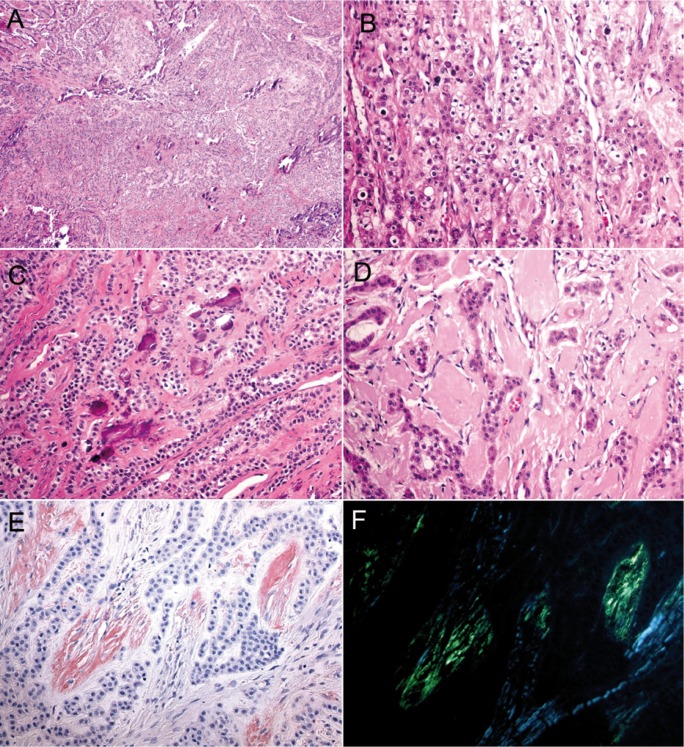
Microscopical findings of clear cell variant of calcifying epithelial odontogenic tumor. A, B) – Nests of polyhedral eosinophilic epithelial cells compounded by large and dark nucleus and clear cells with foamy and clear cytoplasm (H&E, A x25; B x100). C) – Nests of polyhedral epithelial cells with clear cytoplasm and calcification (H&E, x100). D) – Epithelial sheets of polyhedral eosinophilic odontogenic cells surrounded by amyloid deposition (H&E, x100). Amyloid material stained by Congo red E) and exhibited the characteristic green birefringence under polarized light in a darkened background F) (Congo red stain, x100).

**Figure 3:**
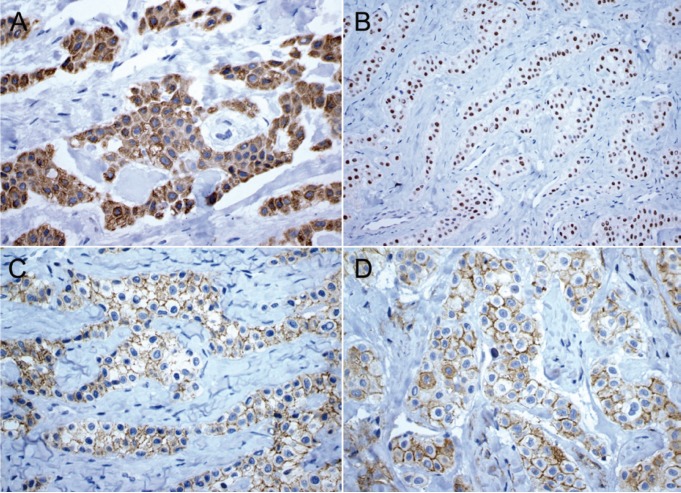
Immunohistochemical findings of clear cell variant of calcifying epithelial odontogenic tumor. Sheets of polyhedral odontogenic epithelial cells positive for CK14 A), p63 B), β-catenin C), and CD138 D) (Immunoperoxidase, x200).

A 25-year-old female patient was referred to the Oral Diagnosis service in Fortaleza (Ceará/Brazil) to evaluation of an asymptomatic nodule in the premolars region of the mandible lasting two months. Extra-oral examination did not reveal changes in the oral and maxillofacial region. Intra-oral examination exhibited an intraosseous swelling of the buccal cortical in the left premolar region of the mandible. Panoramic radiograph revealed a well-defined unilocular radiolucency between left canine and first pre-molar of the mandible measuring 3 x 2 cm, which produced anterior displacement of the canine roots. Radiopaque foci within the lesion and resorption of adjacent teeth roots were not observed (Fig. 1). The radiographic differential diagnosis of the lesion included lateral periodontal cyst, glandular odontogenic cyst, early-stage ossifying fibroma, central odontogenic fibroma, and extra-follicular odontogenic adenomatoid tumor. Under local anesthesia, the patient was submitted to an excisional biopsy through simple enucleation followed by bone curettage. Microscopically, tumor presented strands of polyhedral eosinophilic cells with well-defined borders, distinct intercellular bridges, clear cytoplasm and nuclei pleomorphism interspersed with focal areas of amyloid deposition and presence of irregular calcified structures. The amyloid material was positive for Congo red staining, which exhibited apple–greenish birefringence under polarized light analysis (Fig. 2). The final diagnosis was of clear cell variant of calcifying epithelial odontogenic tumor. The tumor cells were also immunopositive for AE1/AE3 (AE1/AE3, 1:500, Dako), CK14 (LL 002, 1:200, Novocastra), and focally for CK19 (RCK 108, 1:200, Dako) in a cytoplasmic pattern; β-catenin (17 C 2, 1:200, Novocastra) and CD138 (My 15, 1:100, Dako) showed positivity in membrane pattern; and virtually all tumor cells were positive for p63 (4A4, 1:300, Dako) in a nuclear pattern (Fig. 3). The Ki-67 (MIB-1, 1:100, Dako) index was 2%. After 24 months of follow-up, the patient does not present any clinical or radiographic evidence of recurrence.

## Discussion

Although considered uncommon, the presence of numerous clear cells in CEOT has been largely recognized in the literature since the report of the first two cases of this variant by Abrams and Howell in 1967 (3). Since then, less than 40 cases of clear cell variant of CEOT have been documented in the literature so far (2-6). Although some authors have claimed that CEOT composed mainly by clear cells might progress in a more aggressive clinical course with increased rate of recurrence, the proper clinical relevance regarding its biological behavior remains a subject of controversy (4-6,8).

Most patients with CEOT are in their third to fifth decades of life and have experienced a well-defined and circumscribed intraosseous tumor in the posterior region of the mandible that radiographically appears as a unilocular or multilocular radiolucent lesion containing radiopaque structures that is usually associated with unerupted teeth and dental resorption (1,7). The clinical profile of patients with clear cell-rich CEOT is not different from those with conventional CEOT (1-8). The current case exhibited a radiographic appearance of unilocular lesion without radiopaque foci, leading us to consider other odontogenic cysts and tumors in the differential diagnosis.

Microscopically, CEOT is characterized by variable amounts of epithelial, amyloid, and calcifying components (7). CEOT is typically composed by eosinophilic cells with pleomorphic nuclei, regular borders and prominent intercellular ridges, which might show clear appearance due intracytoplasmic accumulation of glycogen (4-6,8). Variable amounts of Congo red-positive amyloid material and irregular or regular concentric (Liesegang ring) calcified formation are present next to the epithelial cells (7). In the current case, the epithelial component predominated when compared with focal areas of amyloid deposition and irregular dystrophic-like calcification. Interestingly, more than half of total quantity of epithelial cells exhibited unequivocal clear cytoplasm, leading us to render out the diagnosis of clear cell variant of CEOT.

The microscopic differential diagnosis of CEOT predominantly composed by clear cells include central mucoepidermoid carcinoma, metastatic malignancies originating from kidney, thyroid, and lung, or other odontogenic tumors such as ameloblastoma and clear cell odontogenic carcinoma (4-6,8). The lack of clinical and radiographic evidence of malignant disease, the absence of microscopic ameloblastomatous differentiation, and the unequivocal presence of amyloid material and calcified formation in the present case were essential features to establish the final diagnosis of clear cell predominant CEOT.

The immunohistochemical findings of clear cell variant of CEOT have been reported in few studies (3,5,7). Tumor cells are immunopositive for different subtypes of cytokeratins (CKs), CK AE1/AE3, CK7, CK8, CK13, CK14, CK19 and negativity for CK10 and CK20 (3,5,7). CEOT with clear cells might also be positive for CD1a and negative for S-100, muscle specific actin, desmin, and antihuman melanosome (3,7,8). Indeed, clear tumor cells showed positivity for CK5, CK19, CD138, and E-cadherin (7). In the current case, all tumor cells were immunopositive for AE1/AE3, CK14, CK19, β-catenin, CD138, p63, with a low index of Ki-67, an immunoprofile compatible with those published in the literature.

 In summary, we report an additional case of clear cell variant of calcifying epithelial odontogenic tumor showing positivity for AE1/AE3, CK14, CK19, β-catenin, CD138 and p63.
